# NMR Characterization of Lignans

**DOI:** 10.3390/molecules27072340

**Published:** 2022-04-05

**Authors:** Roberto Consonni, Gianluca Ottolina

**Affiliations:** Institute of Chemical Sciences and Technologies “Giulio Natta”, National Research Council, Via Corti 12, 20133 Milan, Italy; gianluca.ottolina@scitec.cnr.it

**Keywords:** NMR, aryltetralin, arylnaphtalene, dibenzylbutyrolactone, lignans, *Linum*

## Abstract

Lignans are particularly interesting secondary metabolites belonging to the phenyl-propanoid biosynthetic pathway. From the structural point of view, these molecules could belong to the aryltetralin, arylnaphtalene, or dibenzylbutyrolactone molecular skeleton. Lignans are present in different tissues of plants but are mainly accumulated in seeds. Extracts from plant tissues could be characterized by using the NMR-based approach, which provides a profile of aromatic molecules and detailed structural information for their elucidation. In order to improve the production of these secondary metabolites, elicitors could effectively stimulate lignan production. Several plant species are considered in this review with a particular focus on *Linum* species, well recognized as the main producer of lignans.

## 1. Introduction

The use of metabolomics is spreading widely and includes an increasing field of applications, spanning from the life sciences [1,2] to plant metabolites content [3,4], food science [5,6], natural products [7], up to advanced materials. Novel technologies in instrumental analytics are increasingly [8,9] improving to lower detection limits and the range of detectable substance classes is expanding, broadening the scope of accessible and potentially bioactive natural molecules. The potentiality of metabolomics in the field of human health is well known [10], with the study of biofluids. Most “omics-driven” studies provide considerable amounts of knowledge that can be used in the development of synthetic biology tools and the advancement of metabolic engineering. These studies facilitate the manipulation of complex biological systems toward establishing robust industrial biomanufacturing platforms [11]. The new trend reinforces the use of multi-omics technologies in providing new orchestration towards the improvement of knowledge for comprehending biological processes.

Although metabolic profiles are well suited to capture the host state, most metabolomics studies are down powered, measuring only a restricted set of metabolites in order to reduce the complexity of the chemistry. The use of chemometrics provides valid statistical tools to perform classification and prediction models based on the metabolite profiles [12]. This will critically influence future success and affect the natural product field in pharmaceutical, nutritional, and agrochemical applications and especially in anti-infective research, as in the current pandemic era [13]. In the case of interest in specific classes of chemical compounds, selection of those compounds by means of chromatographic techniques would require a further structural characterization by NMR spectroscopy, using different bidimensional and heteronuclear experiments. This is the case of lignans from plants, in which particular interest in their applications has been shown.

The aim of the present review is to present studies about the potential content of bioactive natural compounds in plants, to reveal tissue-specific accumulation of metabolites. In particular, aryltetralin, arylnaphtalene, and dibenzylbutyrolactone lignans have been investigated in different plant species by means of NMR spectroscopy. A particular focus will include *Linum* species, investigated in different tissues such as callus, hairy roots, and adventitious roots, aimed to characterize and increase the production of lignans.

## 2. Lignans from Plants of Different Species

**Scheme 1:**
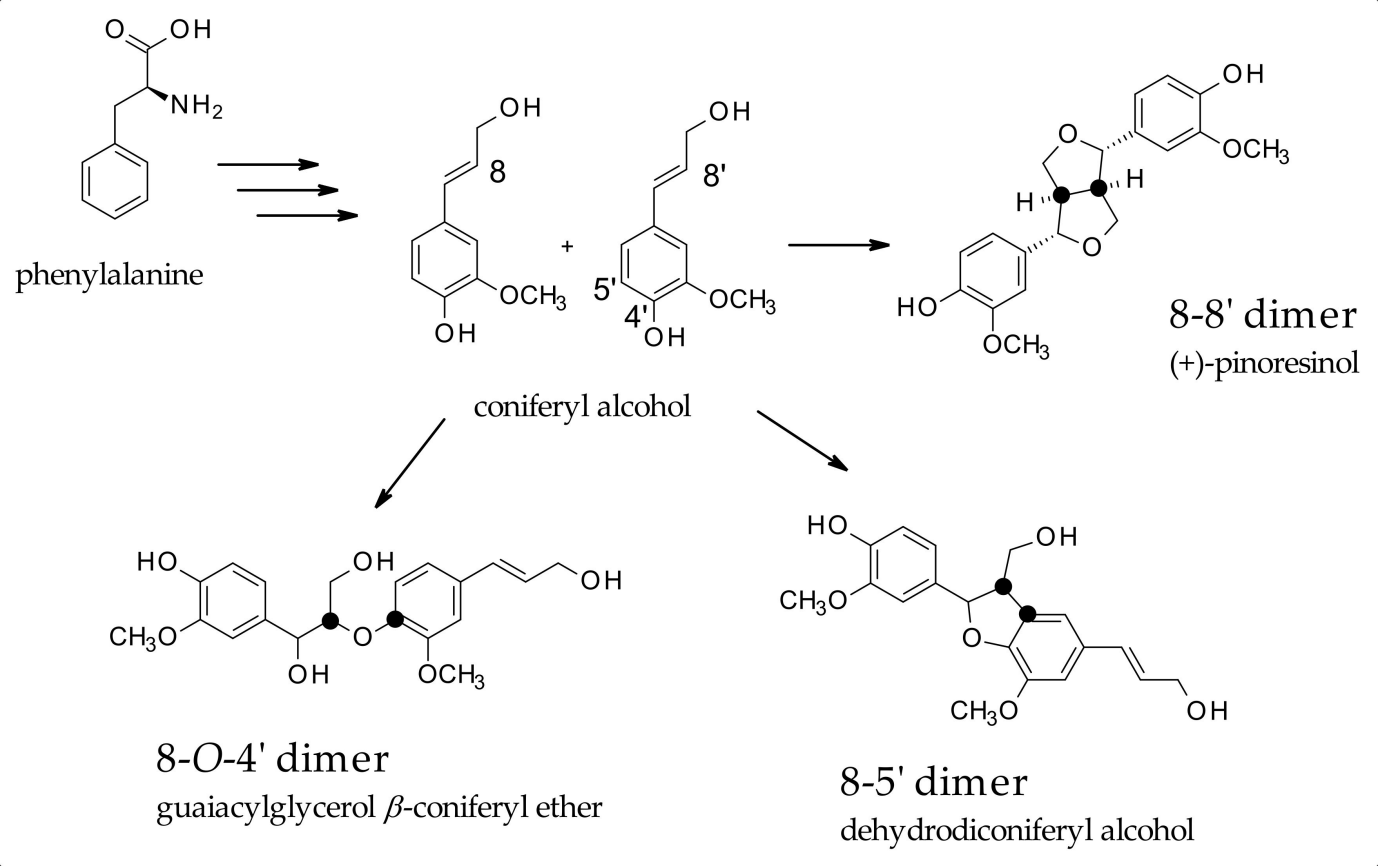
Schematic representation of the formation of lignans (8-8′ dimers) and neolignans (8-*O*-4′ or 8-5′ dimers).

Lignans are classified as phenolic secondary metabolites of plants and are derived from the phenylpropanoid biosynthetic pathway, also known as monolignol-derived dimers. Traditionally, lignans are classified into two main classes, lignans and neolignans. Classic lignans are grouped into other six subtypes, consisting of arylnaphthalene, aryltetralin, dibenzylbutane, dibenzylbutyrolactone, tetrahydrofuran, and furofuran. Conversely, neolignans are more differentiated and consist of fifteen subtypes. Essentially, lignans are derived from the oxidative dimerization of two molecules of coniferyl alcohol (CO) forming an 8,8′ bond, while neolignans are referred to as a different type of linkage, such as 8-5′, 8-*O*-4′, and 8-2′ [14], as represented in Scheme 1.

**Scheme 2:**
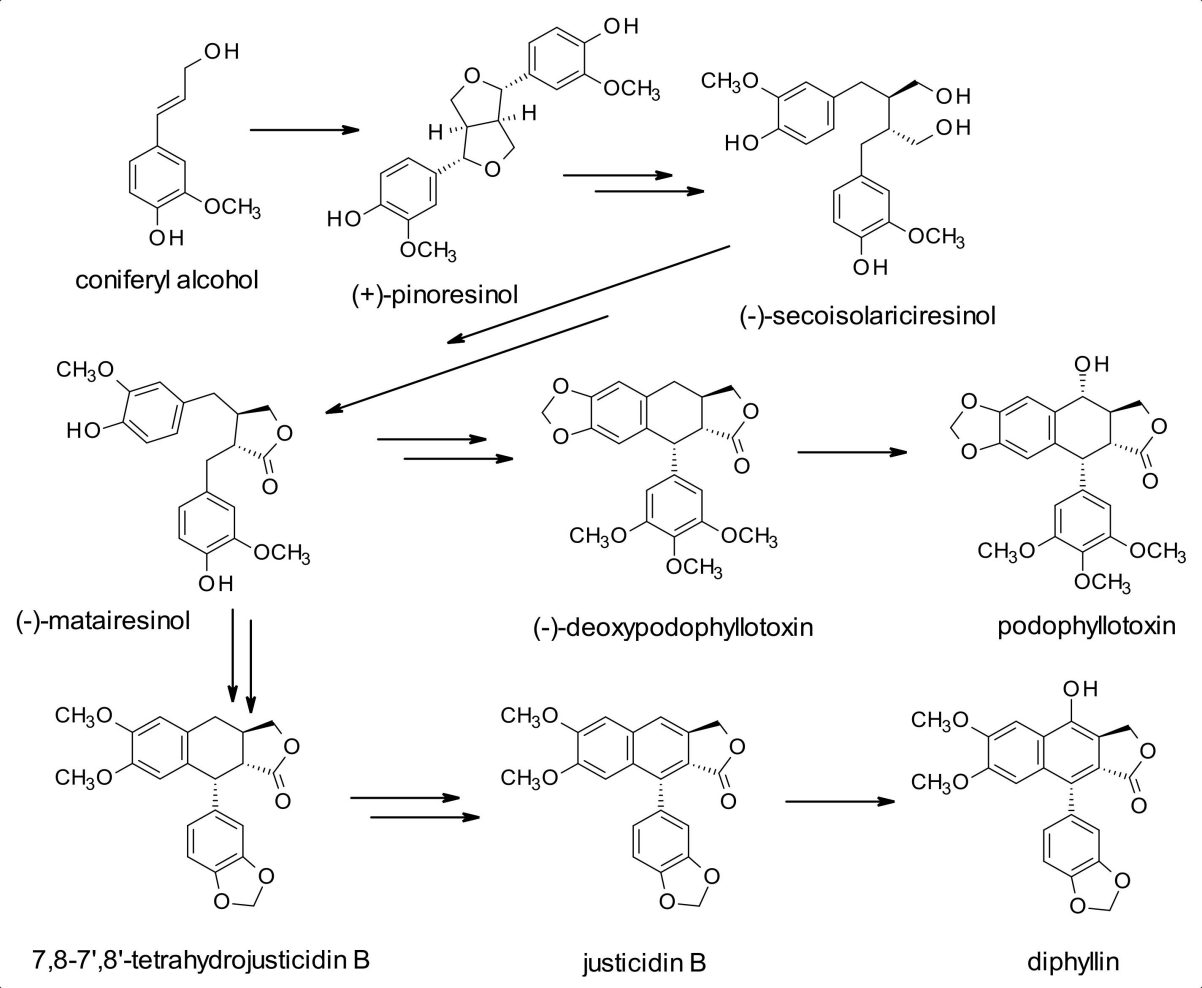
Schematic representation of the biosynthetic pathway of aryltetralin and arylnaphthalene lignans.

The increasing interest in these derivatives is based on their health benefits in terms of antioxidant, antitumor, anti-inflammatory, and antiviral properties, even though the role of these molecules in plants is still not fully elucidated. They have been suggested to play a potential role in stress response mechanisms. Their biosynthesis starts with the coupling of two coniferyl alcohol (CO) residues, leading to the formation of pinoresinol (PR), and through the formation of secoisolariciresinol (SE), and matairesinol (MA) (dibenzylbutyrolactone lignan). This molecule is believed to be a central intermediate leading to different classes of lignans. The biosynthetic pathways could be directed to the production of arylnaphtalene-type lignans, such as justicidin B (JU), and diphyllin (DI)-based derivatives or to aryltetralin-type lignans, such as podophyllotoxin-based derivatives (Scheme 2). These molecules are extracted from various parts of different plant species (bark, root, leaf, flower, fruit, and seed) belonging to different families (Berberidaceae, Linaceae, Rutaceae, Apiacee, etc.), even though flaxseed represents the highest producer of lignans, estimated to be 60–700 times more abundant than other plants. Due to their differentiated employment as bioactive molecules, elicitation, rather than total synthesis procedures [15,16], has been applied in the view of a larger production procedure of compounds, but also to use them as a reference for structural characterization approaches. A subfamily of these compounds is made by dilignans, an uncommon subset of naturally occurring plant products. They have been isolated in different structures, including tetrahydrofuran rings, such as 1 1,4-dioxanes, Diels−Alder products, and lactol dimers. Attempts at total synthesis are also present for these compounds [17]. The neolignans subgroup is widespread in nature as well, with a broad spectrum of biological and pharmacological activities. In this review, NMR structural characterization of lignan is considered for the three main classes: dibenzylbutyrolactone, arylnaphtalene, and aryltetralin structures.

### 2.1. Dibenzylbutyrolactone Lignans

Traditional Chinese medicine has been using extracts from different medicinal plants for a long time. Different spontaneous plants and herbs are used to cure different pathologies, mostly related to inflammatory processes, being extracts rich in polyphenols and lignans, whose biological activities are well recognized. Among those plants, the stems and leaves of *Trachelospermum jasminoides* (Lindl.) Lem are enriched with lignans, flavonoids, and triterpenoids, endowed with several bioactivities in human health. The alcoholic extracts (80%) showed the presence of 28 compounds, including 18 dibenzylbutyrolactone lignans. The extract revealed a moderate inhibitory activity on the NF-KB signaling pathway induced by TNFα. The compounds constituting the extract were found to be trachelogenin, nor-trachelogenin, MA, two newly isolated compounds including MA-4′-*O*-β-d-cellobioside and (*7R,8S*)-dihydrodehydro-CO, isolated for the first time from *T. jasminoides*. The structural characterization of these new compounds was achieved by means of COSY, ^13^C decoupled spectrum, and HMBC experiments. In particular, the cellobioside moiety was assigned by comparing NMR signals with the known resonances of 5-methyl-coumarin-4-cellobioside. According to the author’s findings [18], the cellobioside dibenzylbutyrolactone lignin was first identified, while the CO derivative was confirmed by comparison with the already reported spectroscopic data [19].

Previous work by Fonseca [20] investigated the benzene extract of the knot of dead trees of *Araucaria angustifolia* O. Ktze (pinheiro do Paranà) exposed to water for a long time. Purification by simple silica gel chromatography allowed for obtaining a mixture of representative members of lignans: pinoresinol-di-methyl ether, SE, lariciresinol, isolariciresinol, and mono-methyl ether-isolariciresinol, not described yet in the literature. Those compounds have been characterized by ^13^C-NMR spectroscopy of samples dissolved in chloroform, without the use of multidimensional experiments, showing the capability of the monodimensional carbon data in solving structural tasks.

A recent contribution in the characterization of lignans from the *A. araucana* plant, growing in Chile over 2000 m, is due to Bravo-Arrepol [21]. The knots (picoyo) of this tree are made of extremely hard wood, and with respect to *A. angustifolia* knots enriched in lignans; the latter has not been well characterized. In their study, the authors obtained the phytochemical characterization of heartwood, branches, and knots of *A. araucana*, on the obtained organic extraction from different parts. The quantification of lignans was performed by HPLC and GC-MS analysis, identifying SE, lariciresinol, and MA; while eudesmin, isolated from knots, was characterized by NMR spectroscopy. Interestingly, the authors found remarkable differences in the content of lignans according to different parts of the plant material: knots resulted in the most enriched one, followed by branchwood and stemwood, showing SE as the main lignan (45.77 mg/g), eudesmin (22.68 mg/g), lariciresinol (4.57 mg/g), and MA (1.19 mg/g). In fact, knots were found to have the highest antioxidant activity in terms of DPPH assay, while eudesmin did not display this activity. Again, the cytotoxic activity against SHSY5Y neuroblastoma and P3X myeloma cell lines revealed a moderate activity of the extract in general, but not eudesmin.

*Amischotolype hispida* (A.Rich) nD.Y.Hong (Commelinaceae) is a perennial herb, distributed in different locations, such as the southern part of China (Ryukyus), Indonesia, Papua New Guinea, and Taiwan. The phytochemistry and the biological features have still not been investigated even though there are 15 species of the *Amischotolype* genus worldwide and only one grows in Taiwan. The methanol extract of the whole plant revealed the presence of one new lignan, along with other compounds endowed with antimycobacterial activity. This new lignan was characterized by means of ^13^C-NMR, DEPT, HMBC, NOESY, HSQC, and selective 1D-NOE irradiation experiments, giving rise to the structure of (3*S*,3a*S*,6*S*,6a*R*)-tetrahydro-3-(4-hydroxy-3,5-dimethoxyphenyl)-6-(3-hydroxy-4-methoxyphenyl)-1*H*,3*H*-furo[3,4-*c*]furan-1-one, and named amislignol [22].

Pine (*Pinus silvestris* L.) bark extracts are known to have strong antioxidant and antimicrobial activities, due to the presence of polyphenols, but earlier studies have shown the presence of ferulic acid, pinoresinol, MA, and a novel dihydroflavonol [23]. The aqueous/acetone extract of bark allowed the characterization of three lignans: 1-(4-hydroxy-3-methoxyphenyl)-2-[4-(3-hydroxypropyl)-2-methoxyphenoxy]-propane-1,3-diol, 4-[3-hydroxymethyl-5-(3-hydroxypropyl)-2,3-dihydrobenzofuran-2-yl]-2-methoxyphenol, and pinopalustrin. This result was achieved by Sinkkonen [24] by using the combination of different NMR experiments, such as HSQC, HMBC, DQF-COSY, and NOESY. These lignans were previously described; however, the published NMR results were incomplete.

*Sambucus williamsii* Hance (Coprifogliaceae) is widely distributed in the north of China, Korea, and Japan. This plant has been used for thousands of years in China for the treatment of bone fractures, rheumatoid arthritis, gout, Kashin–Beck disease, inflammation-related gastrointestinal disorders, and kidney disease. Investigation of bioactive compounds of stems and branches of *S. williamsii* allowed the isolation and characterization of four new lignans [25], together with other nine of them already known but reported for the first time in the *Sambucus* genus. The four new lignans were: (7*S*,8*R*)-dihydrodehydro-di-CO 9′-*O*-β-D-apiofuranosyl-(1→6)–*O*-β-d-glucopyranoside (samwiside); 2,10-dimethoxy-8-hydroxypropyl-benzofuran [3,2-c][1]benzopyran (samwinol); (7*R*,8*R*,8′*R*)-4′-guaiacylglyceryl-Evofolin B; (7*R*,8*S*,7′*R*,8′*S*,7″*S*,8″*R*)-4′-guaiacylglyceryl-medioresinol-4-*O*-β-d-glucopyranoside (samsesquinoside). The combined use of HR-ESI-MS and multidimensional/multinuclear NMR spectroscopy achieved the structural characterization [26].

**Figure 1:**
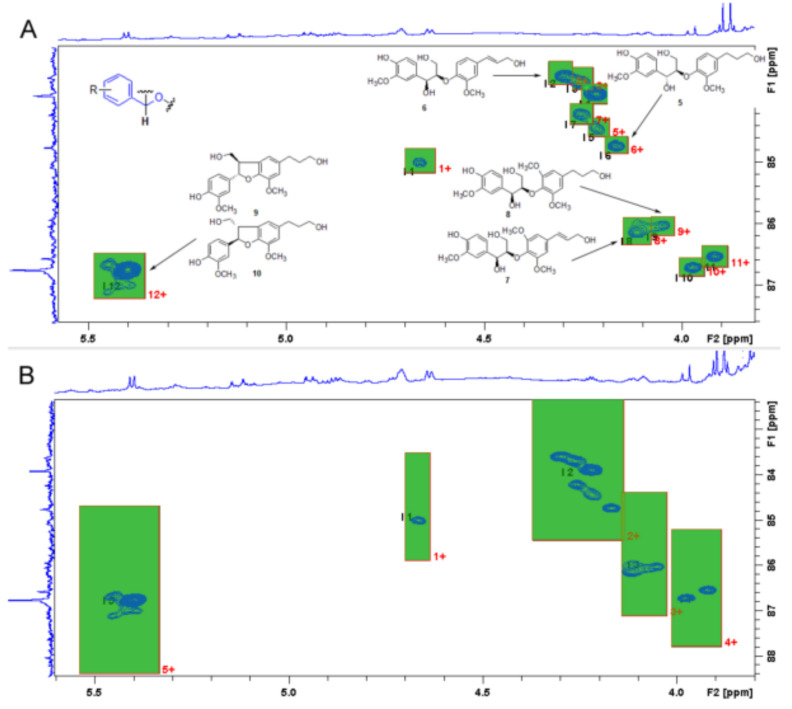
Integral region chosen for active lignan quantitation in SWCA in the ^13^C-NMR spectrum between 80 and 90 ppm using the selective HSQC spectrum. (**A**) Integrating spectra with 12 regions; (**B**) integrating spectra with five regions. Names of the represented compounds are as follow: n° 5, (7R,8R)-1-(4-hydroxy-3-methoxyphenyl)-2-[-4-(3-hydroxypropanyl)-2-methoxyphenoxy]-1,3-propanediol; n° 6, (7S,8R)-guaiacylglycerol-β-*O*-4′-conifery ether; n° 7, (7S,8R)-guaiacylglycerol-β−*O*-4′-sinapyl ether; n° 8, (7S,8R)-1-(4′-hydroxy-3′-methoxyphenyl)-2-[4″-(3-hydroxypropyl)-2″,6″-dimethoxyphenoxy]propane-1,3-diol; n° 9 and 10, 2,3-dihydro-2-(4-hydroxy-3-methoxyphenyl)-7-methoxy-5-(3-methoxy-1-(propenyl)-3-benzofuranomethanol. Reproduced with permission from the original article [27].

Recently, Xiao and coworkers [27] proposed an NMR approach based on the use of the HSQC experiment to estimate the total amount of lignans in a complex mixture. To test this approach, an enriched lignan ethanol extract from the stem and branches of *S. williamsii* Hance (North China red elder) was used. Chromatographic separation of the ethanol extract of this plant showed the presence of thirty-four lignans, several phenolic acids, and triterpenoids [28]. The approach was proposed to set up a reliable method for measuring the level of a bioactive mixture of compounds in a complex extract. Particularly, an NMR quantitative analysis of the amount of lignans in a lignan-enriched fraction was achieved. The proposed method was based on the HSQC experiment, acquired with selective excitation of the ^13^C region (80–90 ppm) where hydroxyl or oxy-benzylic oxygenated secondary carbon occurs, thus characterizing a single lignan structure of this type. Integration of resolved cross-peaks found in this spectral region was referenced to those of a pure standard lignan compound (pinoresinol in the present case) to establish the amount of lignans in the lignan reach mixture. The advantage of using selective HSQC experiments with a Non-Uniform Sampling acquisition mode (NUS) has been compared with the standard HSQC sequence, allowing higher resolution, faster experimental time, and reliable quantification. In particular, the selected carbon region contains only oxygenated benzylic carbons, while the proton region corresponds directly to attached protons, thus increasing the spectral resolution and “filtering” selectively specific moieties. The limitation of this approach is due to the requirement of a hydroxyl/oxy-benzyl lignan moiety (Figure 1).

### 2.2. Arylnaphtalene Lignans

*Phyllantus Flexuosus* (Euphorbiaceae) is a widespread shrub found in tropical and subtropical territories, with about 30 species in South China. Several species have been used as medicinal plants in traditional medicine because of the large amount of bioactive compounds [29]. In the work by Zhao [30], three arylnaphtalene glycosylated lignans (Phyllanthusmin C, arabelline, and diasyringaresinol) have been identified from roots, together with two limonoids (flexuosoids A and B). NMR experiments, such as ^1^H-^1^H homocorrelated COSY, ROESY combined with the hetero-correlated ^1^H-^13^C HMBC experiment, led to the complete resonance assignment of aglycone and saccharide moieties of the methanol extract of the air-dried roots.

*Haplophyllum buxbaumii* is a yellowish-green perennial herb widespread in northern Jordan, but several species are spread throughout the world. There are several species of *Haplophyllum* worldwide*: H. acutifolium* (DC.) G. Don. *H. buxbaumii* (Poiret) G. Don, *H. buxbaumii* (Poiret) G. Don, subsp. Buxbaumii, *H. cappadocium* Spach, *H. glabrinum*, *H. hispanicum* Sprach, *H. myrtifolium* Boiss., *H. patavinum* (L.) G. D. ON. fil., *H. perforatum* (M.B.) Vved., *H. ptilostylum* Spach, *H. suaveolens* (DC.) G. Don, *H. telephioides* Boiss., *H. thesioides* (Fisch ex DC.) G. Don, *H. tuberculatum* (Forssk.) A. Juss., and *H. vulcanicum* Boiss. & Heldr. were the most studied and various compounds have been isolated, including lignans. From this plant, several lignans have been isolated and characterized in the past from the whole plant. In the present work, Al-Abed and coworkers [31] established the structural characterization of two isolated new lignans, namely daurinol glucoside and mono-O-acetyldaurinol glucoside (6-*O*-(β-d-glucopyranosyloxyl)-7-methoxy-l-(3′,4′-methylenedioxyphenyl)-3-hydroxymethylnaphthalene-2-carboxylic acid lactone and 6-*O*-(acetyl-β-d-glucopyranosyloxy)-7-methoxy-1-(3′,4′-methylenedioxyphenyl)-3-hydroxymethylnaphthalene-2-carboxylic acid lactone, respectively), by using COSY and heteronuclear COSY, DEPT, and ^13^C decoupled NMR experiments in organic solution (chloroform). Successively, the same group, from *H. buxbaumii* A. Juss. (Rutaceae) isolated four glucoside-DI, three of which are new lignans [32]. All compounds were investigated in methanol solution by using ^1^H, ^13^C decoupled, and DEPT NMR experiments. The DI derivatives were cleistanthin B, majidine (4-*O*-(β-dxylopyranosyl(1→2)β-d-apiofuranosyloxy}-6,7-dimethoxy-l-(3′,4′-methylenedioxyphenyl)-3-hydroxymethylnaphthalene-2-carboxylic acid lactone), qudsine (4-*O*-β-d-glucopyranosyl(1→3)α-L-arabinopyranosyl(1→5)(2-*O*-acetyl-3-d-apiofuranosyloxy]-6,7-dimethoxy-1-(3′,4′methylenedioxyphenyl)-3-hydroxymethylnaphthalene-2-carboxylic acid lactone, arabelline (4-*O*-α-L-arabinopyranosyl(1→6) β-d-glucopyranosyloxyl-6,7-dimethoxy-1-(3′,4′-methylenedioxyphenyl)-3-hydroxymethylnaphthalene-2-carboxylic acid lactone).

*H. patavinum* L. (Rutaceae) is an endangered plant, whose shoot cultures were investigated in the view of a regeneration protocol, identifying alkaloids such as furoquinoline and quinolone [33]. Contextually [34], methanolic extracts of shoot cultures allowed to isolate a new arylnaphtalene lignin glycoside, namely patavine, not detected in the native plant, in addition to another five known lignans, being JU, DI, tuberculatine, majidine, and arabelline also detected in the aerial parts of the native plant extracts. Also in this study, the combined use of NMR experiments such as COSY, HSQC, and HMBC allowed the structure elucidation of lignans and the sugar moieties, with patavine being characterized by a 4-*O*-β-apiofuranosil-(1→4)-*O*-β-xylopyranosil-(1→2)- apiofuranosil moiety bonded to the diphillin structure.

Extracts of aerial parts of *H. villosum* (a perennial herb from Iran) are used in traditional medicine for different treatments, including testicular cancer, malaria, etc. A previous work on the metabolite content highlighted the presence of lignans, along with alkaloids and cumarins [35]. In a relatively recent work, Parhoodeh [36] elucidated the structure of eudesmin (most likely for the first time), the 4,8-diaryl-3,7-dioxobicyclo-(3,3,0)-octane lignin, by using NMR experiments such as ^13^C decoupled, HSQC, COSY, and HMBC on methanol extracts of aerial parts.

The whole plant extract from *Justicia procumbens* L. (Acanthaceae) has been used in traditional medicine in China, thanks to its activities against fever, laryngopharyngeal swell, and cancer. Previous studies [37] established the presence of ten diarylbutane lignans, twelve arylnaphtalide lignans, and seven arylnaphtalide glucosides. Among them, the activity of justicidine A was reported, and three new arylnaphtalide lignans, namely 6′-hydroxy justicidin A, 6′hydroxy-JU, and 6′-hydroxy justicidin C, in addition to four already known lignans, namely neojusticin A, chinensinaphthol methyl ester, iso-DI, and taiwanin C, were described by Yang [38]. The structures of these compounds, obtained as pure compounds after the purification of the ethyl acetate extract of the original ethanol extract of the whole plant, were determined by comparison of MS data and a series of NMR experiments, such as ^13^C decoupled, HSQC, and HMBC. Da Silva [39] proposed the synthesis of three arylnathalene lignans, namely taiwanin C, 4-methyl dehydroretrodendrin, and JU, and produced more accurate NMR data using NOESY difference experiments, ^13^C spectra, G-BIRDR, and CPMG-HSQMBC (this last implemented by the authors), and gradient-enhanced versions of COSY, HSQC, and HMBC experiments. In this study, the most important vicinal coupling constants were also measured. This work followed a previous one from the same group but focused on the synthesis of aryltetralin lignans (see Section 2.3).

The most interesting arylnaphtalene lignan, concerning the pharmacological activities, is JU, isolated from different plant origins, particularly *Justicia*, *Phyllanthus*, *Haplophyllum*, and *Linum* species as the major source of JU. Biological activities such as antifungal, antiviral, antibacterial, antiparasitic, piscicidal, antiplatelet, anti-inflammatory, in vivo animal toxicity, and cytotoxicity have been assessed. Different approaches have been performed towards the total synthesis in order to optimize the synthetic processes for industrial applications and have been summarized and elucidated in a recent review by Hemmati [40]. A detailed structural characterization of JU with the combined use of HPLC and HPLC-MS, high-speed counter-current chromatography (HSCCC), and NMR analysis has been performed.

### 2.3. Aryltetralin Lignans

A new aryltetralin lignan was characterized from the roots of *Kadsura coccinea* (Lem) A.C. Smith (schisandraceae, a subfamily of magnoliaceae) known to be enriched in lignans and triterpenoids [41]. The new lignin, namely heilaohusu E, was characterized by the combined use of MS spectrometry and NMR experiments performed on the purified methanol extract of roots. The extracts of this plant, adopted in Tujia medicine in China, have already demonstrated several biological activities, such as antitumor, anti-HIV, antioxidative, and hepatoprotective effects. Previous determinations revealed twenty-three lignans, of which three new ones bear the dibenzocyclooctadiene skeleton and the other twenty are analogs [42]. With the lignans content being the primary characteristic constituents with various biological activities of plants from the *Kadsura* genus, recently, a review summarized 81 lignans isolated and characterized by NMR over the past eight years (from 2014 to 2021), which belong to five types: dibenzocyclooctadienes, spirobenzofuranoid dibenzocyclooctadienes, aryltetralins, diarylbutanes, and tetrahydrofurans [43], thus providing a useful reference for the structural analysis of lignans. The relationships between lignans and biological activities were also systematically analyzed, together with their pharmacodynamics properties.

Wichers and coworkers [44] investigated cell cultures of *Linum flavum* to evaluate the production of aryltetralin lignans. These molecules showed structural similarities with podophyllotoxin derivatives and are used in the treatment of malignancies such as lung carcinoma and Hodgkin’s disease. In general, roots were the major site for the accumulation of podophyllotoxin derivatives, with over 48% of the accumulation. The occurrence of aryltetralin lignans was also compared in different plant organs, such as leaf, stem, root, and cell cultures. Among those molecules, 6-metoxypodophyllotoxin (MPO) appeared to be the most abundant in cell cultures of *L. flavum*, followed by leaf, stem, and root, as well as its glucoside derivative. The structures of the other lignans were characterized by NMR and defined as NC430, NC414, NC400, NC370, MA, and podophyllotoxin. The synthesis of aryltetralin lignans is always a direct way of obtaining pure compounds in order to use them as reference molecules for resonance assignments of analogs to test them for their activity as single compounds or use them to prepare derivatives. In this view, Da Silva [45] synthesized and fully characterized three intermediates of polygamain, morelesin, and 4,5 dimetoxymorelesin. A full resonance assignment (^1^H and ^13^C) was achieved by means of HSQC, HMBC, and the traditional NOESY difference experiment, and information concerning the stereochemistry was obtained by the evaluation of J_HH_ vicinal coupling constants. 

Klaes [46] isolated a new aryltetralin lignan from the seeds of *L. flavum*, var. compactum, previously detected in the aerial parts of several *Linum* species, and which has not been previously reported. The MPO-7-O-hexanoate was fully characterized by NMR data by means of ^1^H, ^13^C, HSQC, and HMBC experiments.

### 2.4. Monolignols and Other Lignans

As already pointed out, lignans are derived from the oxidative dimerization of CO involving carbon 8 and forming an 8-8′ bond. Neolignans are conversely referred to define all other types of linkages [47]. The mostly found lignans in flaxseed (*L. usitatissimum*) are SE-di-glucoside and MA, endowed with important pharmacological activities in human health, in reducing the incidence of breast and prostate cancers by modulating steroidal hormone synthesis [48]. 

To shed light on the biosynthesis of secondary metabolites, in vitro cultures could be a very useful tool as this system allows uniformity, accessibility, and reduces complexity [49]. To this aim, Beejmohun [14] evaluated the supplement of synthetic double-enriched ^13^C 8,9-coniferin and unlabeled compound to cell growth for 14 days to verify the incorporation and metabolism with the production of different types of lignans. The use of labeled compounds facilitated their identification with both LC-MS and NMR methods. The results showed, first of all, that the cell growth had the same rate, either fed with or without coniferin. In this way, four labeled lignans were identified in cell suspensions fed with coniferin: lariciresinol diglucoside (8-8′-linked lignan), dehydroconiferylalcohol glucoside (8-5′ linked neolignan), and erythro/threo guaiacylglycerol-β-coniferyl-ether-glucoside (8-*O*-4′ linked neolignan), being these last structures first detected in flax.

A convenient semi-synthesis of (8*R*,8′*S*)-matairesinol-4,4′-di-*O*-β-glucopyranoside was proposed by Ekholm [50] giving rise to 28% of yield in six steps. This lignan was observed in the stems of *T. asiaticum* var. intermedium, a medicinal garden plant (Apocynaceae), and selected as the target molecule for the semi-synthesis. In this way, the pure lignan was fully characterized by NMR (monodimensional ^1^H, ^13^C decoupled and TOCSY, bidimensional DQF-COSY, NOESY, HSQC, HMBC experiments) and the resonance assignment was confirmed by the use of PERCH NMR simulation software, to check its potentiality.

## 3. Lignans from *Linum* Species

Lignans are generally more abundant in *Linum* species with respect to other plant species. The *Linum* genus includes more than 200 species taxonomically divided into five or six sections [51,52] and mainly accumulates arylnaphthalene-type lignans. The seeds of *L. usitatissimum* L. (flaxseeds) are known to be a rich source of the polar glycosidic dibenzylbutanediol-type lignan SE-diglucoside (SDG) which is considered a valuable food constituent due to its function as a precursor for enterolignans [53,54]. In fact, the chemopreventive activity against various tumors and cardiovascular disorders [55] of these compounds has been demonstrated. The concentration of lignans in flaxseeds is known to reach 3% (*w/w*) thus making this part of the plant the main source of these molecules. Stuijis and coworkers [56,57] applied a saponification of lignans followed by purification by means of preparative RP-HPLC in order to isolate and characterize ethanolated molecules by NMR and MS/MS analysis. A direct link between glucosides and hydroxylcynnamic acid was detected. Particularly, hydroxy-methyl-glutaric acid resulted in esterification to SDG, shedding light on structural elements involving *p*-coumaric acid glucoside and ferulic acid glucoside in flaxseed hull. Studies on flaxseed capsules of *L. usitatissimum* at five early stages of their development provided a metabolic profile of intermediate lignan biosynthesis [58,59]. The use of ^1^H and ^13^C decoupled and HR-MS NMR analyses resulted in the identification of 6a-HMG (hydroxymethyl glutaryl) SDG and 6a,6a′-di-HMG SDG as the two major components of the ester-linked lignans. Interestingly, on the basis of metabolic tracer analyses, the authors were able to follow the biochemical pathway for these metabolites, precursors for the formation of the SDG-HMG ester-linked oligomers. 

**Figure 2:**
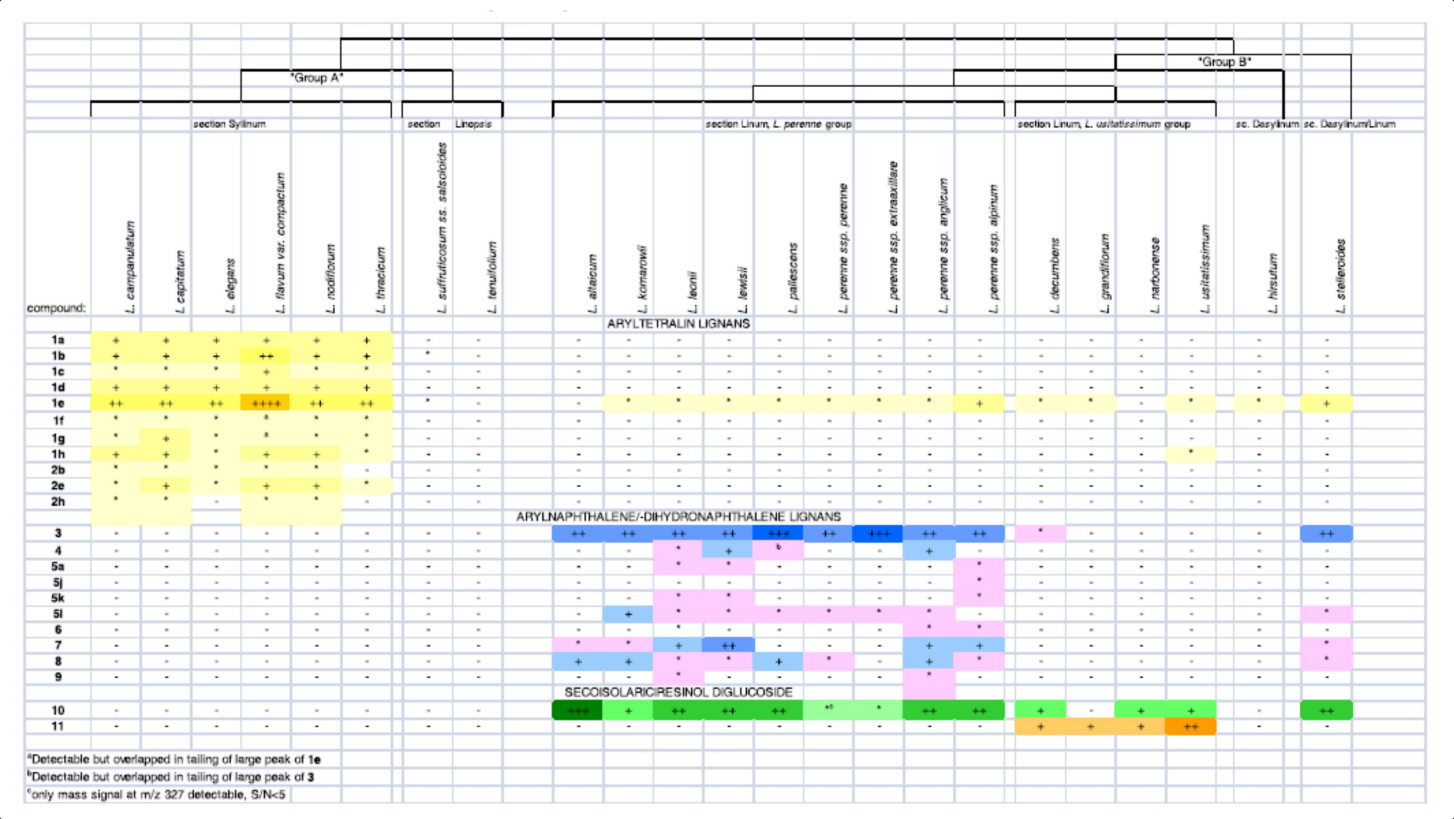
Distribution of lignans in seeds of Linum species. Taxa are ordered according to phylogenetic relationship. Entry symbols represent approximate semi-quantitative estimates of concentration in mg/g seed material;.++++ >5, +++ >3<5, ++ >1<3, + >0.1<1, * <0.1; for details see experimental part. Reproduced with permission from the original article [60].

In the review by Schmidt [60], mature seeds of 20 different varieties of *Linum* were considered, searching for the lignan content (Figure 2). Their result showed SDG in 15 out of 16 seed samples investigated from taxa data (*Linum* and *Dasylinum*), while the seeds of *L. perenne* L., as well as those of several other representatives of sections *Linum* and *Dasylinum*, were found to contain significant concentrations of the arylnaphthalene JU. Other compounds of this type and some aryldihydronaphthalene-type lignans were also present. Contemporarily, the seeds of *L. flavum* and representatives of section *Syllinum* were found to contain aryltetralin-type lignans, mainly in the form of esters with aliphatic carboxylic acids, such as 6-methoxypodophyllotoxin-7-*O*-n-hexanoate, whose occurrence in *L. flavum* seeds has very recently been reported by us for the first time. 

A convenient isolation and purification protocol for SDG was proposed by Chimichi [61] from the flaxseed of *L. usitatissimum*, followed by definitive NMR characterization as 2,3-bis[(4-hydroxy-3-methoxyphenyl)methyl]-1,4-butanediyl bis-[R-(R*; R*)]-β-D-glucopyranoside, by means of ^1^H, ^13^C, DQF-COSY, GE-HSQC, and GE-HMBC experiments. Ten years later, an effective method for obtaining SDG from *L. usitatissimum* seeds was proposed by Stasevich [62]. The process for obtaining SDG includes successive extraction to give a mixture enriched with this compound and its subsequent separation using preparative ion-exchange and reversed-phase chromatography. The proposed method allowed to isolate lignan and SDG with a purity of 95.3% in a 1.03% yield relative to the initial extract. The isolated compounds have been characterized by IR, MS, and NMR analysis.

A localization study of SDG [63] was applied to harvest material from different cell layers of seed coats of mature and developing flaxseed (*L. usitatissimum*) indicating that SDG is synthesized and accumulated in the parenchymatous cell layer of the outer integument of flaxseed coats. NMR and HPLC analysis were used to identify and quantify SDG in dissected cell layers after alkaline hydrolysis. 

A further study [64] investigated the kinetics of the incorporation of phenolic compounds into lignan macromolecules during flaxseed development of *L. usitatissimum* (cv Barbara) by means of HPLC and NMR analysis. The results showed the amount of accumulated free form of monolignol glucosides (9.85 mg/g dry matter) at the early developmental stages. Hydroxycinnamic acid glucosides and flavonoids accumulate in a lower amount and in the later stages of development as esters of the lignan (up to 3.18 and 4.07 mg/g dry matter, respectively). Finally, secosiolariciresinol diglucoside accumulates (up to 28.65 mg/g dry matter) in the later developmental stages as well, in both forms, ester-bound and in free form.

Several groups achieved the lignan structural characterization by extracting them from different plant tissues; others performed chemical synthesis to provide a larger amount of reference materials. It has been reported by several authors that plant cell cultures can overcome the long period of time needed for field cultivation of the plant material. Additionally, metabolite production of these cultures can be enhanced through elicitor treatments [65]. Elicitors are often used to stimulate the production of plant secondary metabolites in diverse species [66]. In particular, elicitors such as methyl jasmonate or coronatine have been reported to enhance lignans production in several *Linum* species. This approach represents a reliable source of bioactive molecules even though some limitations are present, such as the typically low and variable yields of metabolites and genetic instability. The low yield of secondary metabolites in cell cultures has been explained by the lack of cell differentiation. An alternative strategy could be the use of organized tissue such as cultures of roots or shoots [49]. A wide range of natural products has been produced using adventitious and hairy-root cultures, including lignans, by exploiting their high genetic stability, biosynthetic capabilities, and biomass production [67]. These tissues can be cultivated in large-scale bioreactors for commercial production of bioactive compounds and can be stimulated to obtain a higher yield. Until recently, genetically transformed hairy roots had received more attention for phytochemicals production than adventitious roots. However, adventitious roots cultures are emerging since they are considered a more natural material (untransformed), thus leading to their high commercial value compared to hairy roots.

In this view, cell suspension cultures *of L. usitatissimum* (cv Barbara) have been used to produce DCG (dehydrodiconiferyl alcohol-4-β-glucoside) to evaluate the role of this molecule in cell cultures’ defense response. Elicitation with specific fungal pathogens for flax (*Fusarium oxysporum* F. sp. Lini, and *Botritis cirenea*) has been adopted, and the accumulation of DCG has been monitored as a function of time by semi-quantitative reverse transcriptase-polymerase chain reaction. The investigation of the flax cell suspension by HPLC revealed small amounts of lignans, and semi-preparative HPLC allowed to obtain the isolated compound that was fully characterized by NMR experiments (^1^H, ^13^C decoupled, DQF-COSY, HSQC, and HMBC) and MS analysis, establishing the position of the glycosylation [68]. Lignans such as pinoresinol diglucoside and SDG were also detected in the flaxseed of *L. usitatissimum*, in combination with other flavonoids and a new phenylpropanoid glucoside (linusitarnarin) characterized by NMR experiments [69].

In another study, proposed by the Berim group [70], the effect of two elicitors, namely coronalon and indanoyl-isoleucine, were evaluated in cell cultures of *L. nodiflorum*. Their results showed a more than ten-fold increase in the production of MPO. In addition, the production of 5′-demethoxy-podophyllotoxin (5′dMPO) was detected and characterized by NMR experiments. Its average content was evaluated to be over 5% of the cell’s dry weight. Meanwhile, the activities of deoxypodophyllotoxin 6-hydroxylase and β-peltatin-6-*O*-methyltransferase, the enzymes involved in MPO biosynthesis, were increased up to 21.9-fold and 14.6-fold, respectively, in treated cultures. A further evaluation of another elicitor, namely methyl jasmonate, exerted only a moderate stimulating effect on the synthesis of MPO and 5′-dMPO in *L. nodiflorum* cells. 

A relatively recent work by Jullian-Pawlicki [71] used the hairy roots of *L. perenne* to arylnaphtalene and aryltetralin-type lignans. In particular, DI-3-pentose, DI-2-hexose-pentose, and DI-hexose were tentatively assigned for the first time by LC-MS and LC-MS/MS. The aryltetralin 6-methoxypodophyllotoxin and one isomer were also detected in smaller amounts. Finally, two other new molecules were detected, while other aryltetralin-type lignans, such as β-peltatin, were not present in these hairy roots while JU was the main component. The two new molecules were purified by HPLC and the combined analysis performed by MS and NMR, confirmed to be two distereoisomers of 6-methoxypodophyllotoxin (7′α, 8′β-H, 8α-H, 7α-OH, 7′α, 8′α-C, 8β-C,7α-OH and 7′α, 8′α-H, 8α-H, 7α-OH, 7′α, 8′β-C, 8β-C,7α-OH).

A recent paper [72] reported the development of in vitro cell cultures (cells, adventitious roots, and hairy roots) of *L. austriacum* for the production of JU. Cell cultures were also elicited with coronatine and methyljasmonate to stimulate metabolite production. The NMR investigation showed adventitious and hairy roots as the highest productive tissues in terms of justicidine B, iso-justicidine, and an unidentified compound; the elicitors incremented the synthesis of justicidine B more than three times, as well as other aromatic compounds. Additionally, the intracellular localization of justicine B in adventitious roots was proved for the first time. 

Over the last few years, our group has focused on the characterization of lignans from different varieties of *Linum*. In particular, different tissues such as callus, hairy roots, and adventitious roots have been investigated for *L. mucronatum*, *L. bienne*, *L. flavum*, *L. himmelszelt*, and *L. austriacum*. In some cases, preliminary TLC purification has been performed to reduce the complexity of the raw extract.

Extracts from tissues such as roots, callus, and adventitious roots were investigated for *L. mucronatum* by NMR. Roots extracts revealed the presence of CO and pinoresinol, while other tissues revealed the presence of aryltetralin-type lignans mainly pinoresinol, MPO, as well as NC400 and NC414 derivatives, as previously described [44].

Extracts from cell cultures of *L. himmelszelt* revealed the presence of both CO and ferulic acid, while *L. bienne* extracts from cell cultures showed the presence of a coniferyl derivative (most likely coniferin). Extracts from the callus of *L. valoal* were also enriched in coniferine, although in a smaller amount than that detected in *L. bienne*.

Extracts from adventitious roots of *L. flavum* showed the presence of MPO in addition to its glucoside derivative.

**Figure 3:**
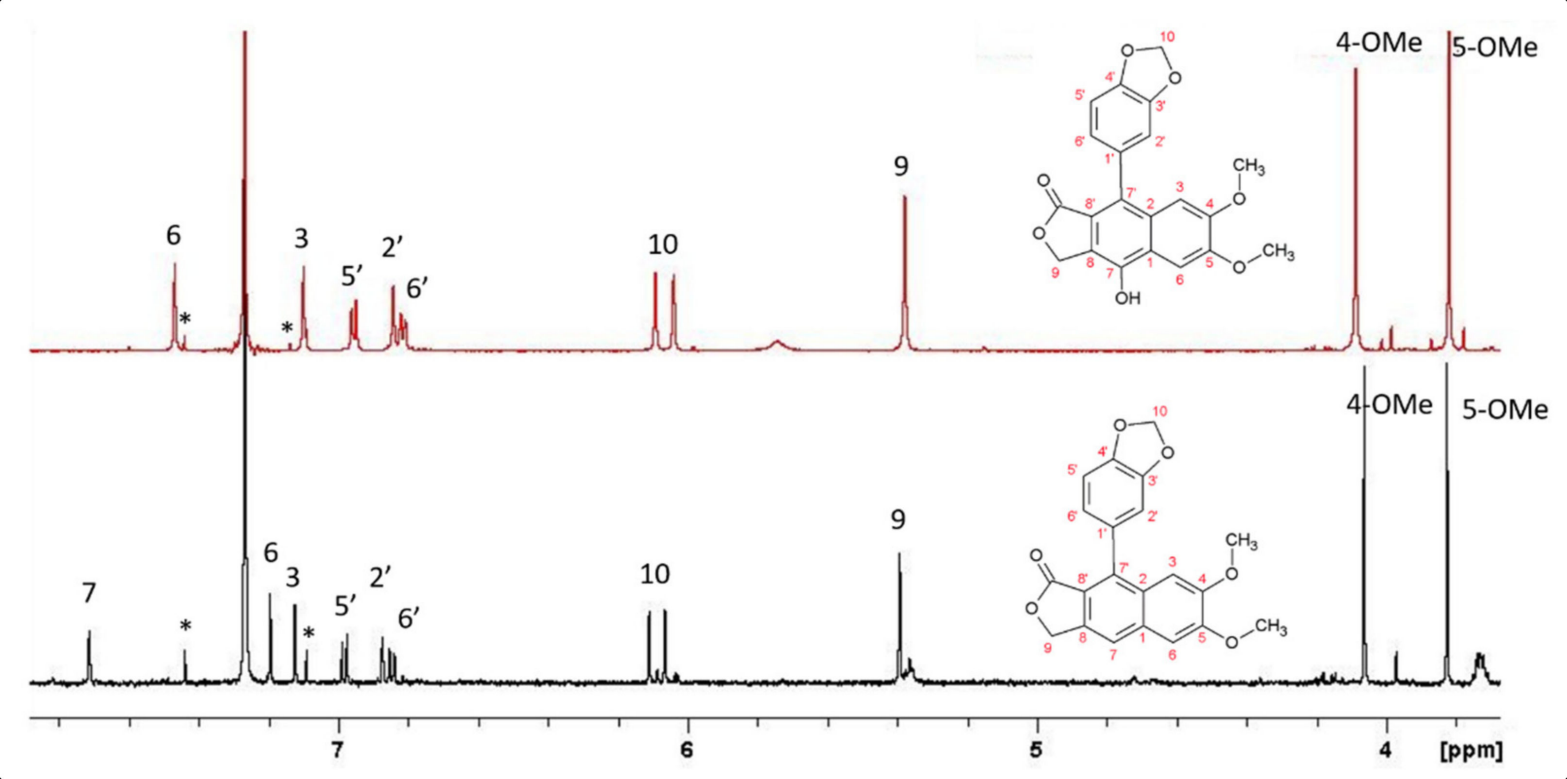
^1^H-NMR spectra recorded at 600MHz of TLC-purified extracts from *L. austriacum* cell cultures containing JU (**bottom**) and DI (**top**) in CDCl_3_ solution. Resonance assignment is reported for clarity. Asterisks indicate ^13^C satellite signals of the solvent.

*L. austriacum* is, among the investigated species, the only species that produces arylnaphtalene lignans. In fact, extracts of the callus revealed the dominant presence of justicidine B. In addition, other lignans belonging to the same family were detected, such as DI and taiwanin, in addition to phenolic derivatives such as coniferyl derivative, tyramine, tyrosine, and cumaric acid (Figure 3).

**Figure 4:**
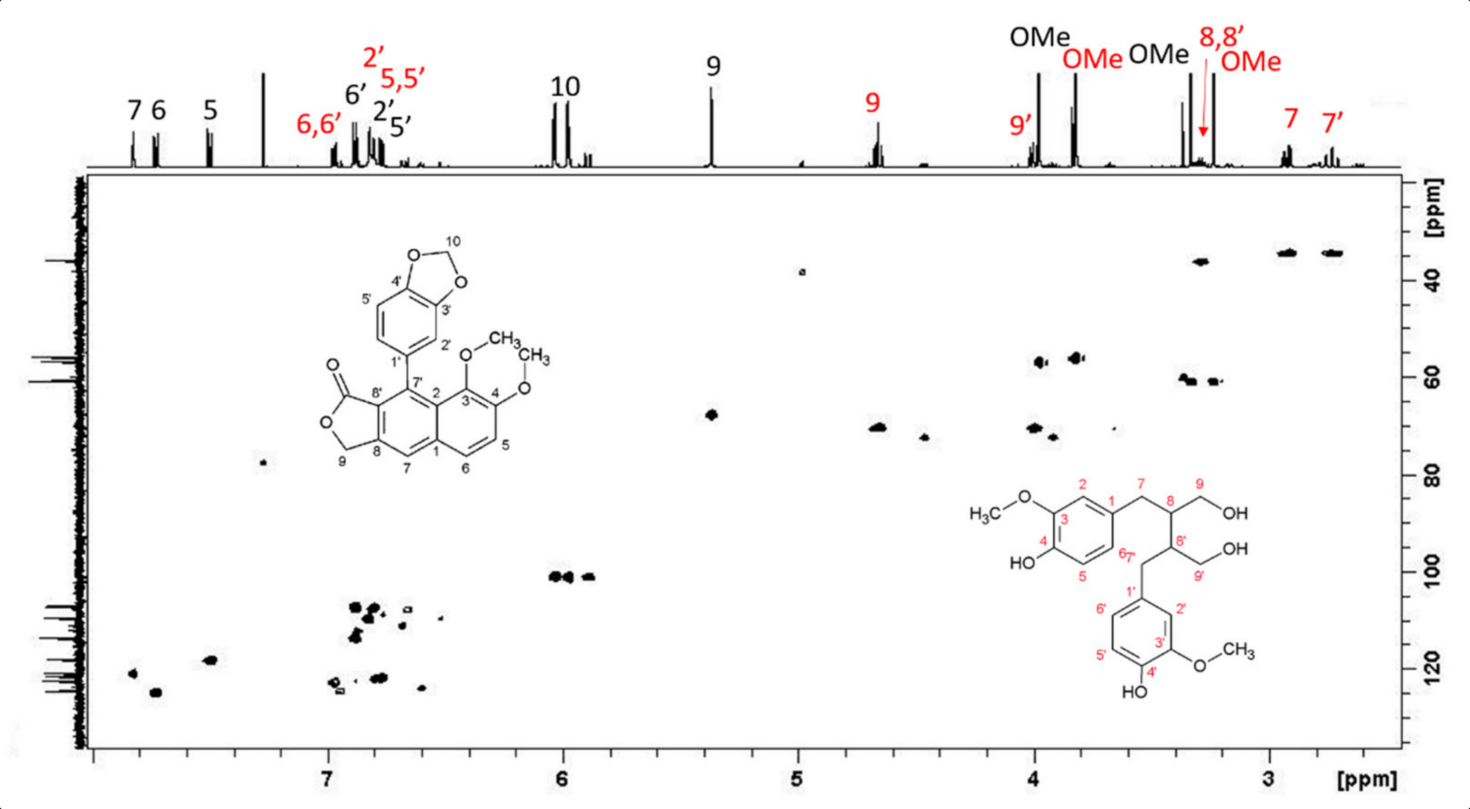
^1^H-NMR spectra recorded at 600MHz extracts from *L. mucronatum* cell cultures containing isojusticidin and secoisolariciresinol in CDCl_3_ solution. Resonance assignment is reported for both compounds (black isojusticin, red secoisolariciresinol. Another unknown compound is present in a much smaller amount and is not yet assigned.

Partially purified extracts from the adventitious roots of *L. austriacum* revealed the presence of two other molecules, present in large quantities and belonging to the arylnaphtalene moiety. The combined analysis of HSQC and HMBC-NMR spectra allowed the identification of isojusticidin and secoisolariciresinol represented in Figure 4 with black and red numbering, respectively.

## 4. Conclusions

Lignans are quite widespread compounds in plants, as representative molecules of the phenyl-propanoid pathway. The interest towards these molecules is driven by their pharmacological actions as antioxidant and anti-inflammatory compounds. Seeds represent the most important reservoir for these molecules and suspension cultures represent a valid profitable and alternative production tool. 

In this respect, cell cultures can be supported in the production of these secondary metabolites by the use of plant growth regulators that initiate or enhance the biosynthesis or by the addition of intermediate molecules of the pathway to the living cell system. Elicitors play many important roles in defense response by acting as signal molecules, thus inducing the production of secondary metabolites. In the present review, the structural characterization by NMR spectroscopy of lignans belonging to the three main classes of dibenzylbutyrolactone, arylnaphtalene, and aryltetralin has been summarized. Looking at the literature, it is quite clear that the common procedure for lignan identification follows the classic route of extraction, possible partial purification, and identification/characterization, preferring this “targeted” approach with respect to the “untargeted” approach. In fact, the metabolomics approach remains the preferred way of investigation when a large number of samples are concerned, and not for single-molecule characterization, particularly when the structural details of unknown compounds need to be elucidated. Lignans have been found in different varieties of plants, particularly in different varieties of *Linum*, the main lignans producer. Cell culture is a feasible approach to producing secondary metabolites, and the elicitor activity is able to activate routes for the production of lignans, even though in some cases only simple antioxidant molecules are accumulated. Among the elicitors, methyljasmonate and coronatine appear to be the most efficient stimulators. This approach suggests possible applications and future scale-up processes to obtain interesting secondary metabolites such as lignans molecules.
